# A Postural Assessment Utilizing Machine Learning Prospectively Identifies Older Adults at a High Risk of Falling

**DOI:** 10.3389/fmed.2020.591517

**Published:** 2020-12-04

**Authors:** Katharine E. Forth, Kelly L. Wirfel, Sasha D. Adams, Nahid J. Rianon, Erez Lieberman Aiden, Stefan I. Madansingh

**Affiliations:** ^1^Zibrio, Inc. Houston, TX, United States; ^2^Department of Internal Medicine, Division of Diabetes, Endocrinology and Metabolism, McGovern Medical School, University of Texas Health Science Center at Houston, Houston, TX, United States; ^3^Department of Surgery, McGovern Medical School, University of Texas Health Science Center at Houston, Houston, TX, United States; ^4^Department of Family and Community Medicine, McGovern Medical School, University of Texas Health Science Center at Houston, Houston, TX, United States; ^5^Department of Internal Medicine, Division of Geriatric and Palliative Medicine, McGovern Medical School, University of Texas Health Science Center at Houston, Houston, TX, United States

**Keywords:** balance, stability, postural stability, fall risk, aging, fall prediction, machine learning

## Abstract

**Introduction:** Falls are the leading cause of accidental death in older adults. Each year, 28.7% of US adults over 65 years experience a fall resulting in over 300,000 hip fractures and $50 billion in medical costs. Annual fall risk assessments have become part of the standard care plan for older adults. However, the effectiveness of these assessments in identifying at-risk individuals remains limited. This study characterizes the performance of a commercially available, automated method, for assessing fall risk using machine learning.

**Methods:** Participants (*N* = 209) were recruited from eight senior living facilities and from adults living in the community (five local community centers in Houston, TX) to participate in a 12-month retrospective and a 12-month prospective cohort study. Upon enrollment, each participant stood for 60 s, with eyes open, on a commercial balance measurement platform which uses force-plate technology to capture center-of-pressure (60 Hz frequency). Linear and non-linear components of the center-of-pressure were analyzed using a machine-learning algorithm resulting in a postural stability (PS) score (range 1–10). A higher PS score indicated greater stability. Participants were contacted monthly for a year to track fall events and determine fall circumstances. Reliability among repeated trials, past and future fall prediction, as well as survival analyses, were assessed.

**Results:** Measurement reliability was found to be high (ICC(2,1) [95% CI]=0.78 [0.76–0.81]). Individuals in the high-risk range (1-3) were three times more likely to fall within a year than those in low-risk (7–10). They were also an order of magnitude more likely (12/104 vs. 1/105) to suffer a spontaneous fall i.e., a fall where no cause was self-reported. Survival analyses suggests a fall event within 9 months (median) for high risk individuals.

**Conclusions:** We demonstrate that an easy-to-use, automated method for assessing fall risk can reliably predict falls a year in advance. Objective identification of at-risk patients will aid clinicians in providing individualized fall prevention care.

## Introduction

Falls are the leading cause of trauma death and trauma admissions (1) in large hospital systems across the US, and the leading cause of accidental death in older adults (2). Each year, 28.7% of older adults fall in the US (3), which results in ~300,000 hip fractures, and over $50 billion dollars in medical costs (4). Yet, despite the dramatic impact falls have on health, fall risk assessments and management were infrequently utilized in primary care (5) until 2011 when the Centers for Medicare & Medicaid Services required fall risk assessments for all Medicare annual exams (6). Despite this requirement, there is no clear gold standard in clinical assessments for fall risk (7).

The challenge for creating a gold standard fall risk assessment is the many contributing risk factors, including cognitive impairment, balance and gait abnormalities, disabilities of the lower limbs, foot problems (8), vision impairment (9), fall history (10), and fear of falling (11). Of these risk factors, fall history is considered the best predictor of falls (10) and forms the basis for the recommended clinical practice guidelines for fall prevention (12). Unfortunately, less than half of patients will actually report falls to their physicians (13). One approach to improving fall risk assessment is to quantify an individual's intrinsic stability control mechanisms using posturography.

Posturography characterizes the sway of an individual's center-of-mass (COM) over time using measures of position, velocity, acceleration and jerk. To date, the resulting measurements have been shown to have modest fall prediction capabilities (14–18), although these have been limited by the difficulties associated with tracking falls in an aging population, resulting in limitations on sample sizes and on the length and quality of follow-up. Other limitations to date include: choices about which fall types are studied [e.g., multiple falls (18), indoor falls (14)], use of complex protocols, use of expensive equipment, and requirements of testing under multiple conditions (18). Characterizing COM as a system which dynamically shifts through non-linear stability states of equilibria may provide deeper insight into balance control and yield greater predictive capability as it will reveal intrinsic postural control failures (19, 20).

In this study, we assessed the validity and reliability of an eyes open, 60 s standing balance test, performed on a commercially available balance platform that automatically calculated a postural stability (PS) score using linear and non-linear stability states, as an indication of fall risk for older adults. Prospective fall risk data were collected in a large, heterogeneous population of older adults to assess overall predictive fall risk performance.

## Methods

### Participant Recruitment

In order to assess the accuracy of fall risk assessments based on a PS score, we recruited 209 community dwelling adults to participate in a yearlong prospective study. These individuals were drawn from eight different independent senior living facilities (tested on site) and five local community centers (tested at the Texas Medical Center Innovation Institute). This prospective cohort was part of a larger clinical trial wherein a total of 412 adults were enrolled. The remaining 203 participants were recruited from physical therapy clinics, geriatric medicine outpatient clinics and a hospital rehabilitation ward in the greater Houston area participated in a cross-sectional study only and were not tracked longitudinally. Individuals who were unable to stand for 60 seconds unassisted, those who self-reported severe vestibular problems (e.g., Meniere's disease) or musculoskeletal issues related to balance control, and those who self-reported a history of dementia and were considered unable to provide accurate fall history due to cognitive deficits were also considered exclusion criteria. Only one person was excluded in the study. The experimental protocol was approved by the Westerns IRB (#20171926 and #20172324), and the University of Texas Health Science IRB HSC-MS-16-0019), and informed consent was obtained prior to testing.

### Data Collection

**Figure 1:**
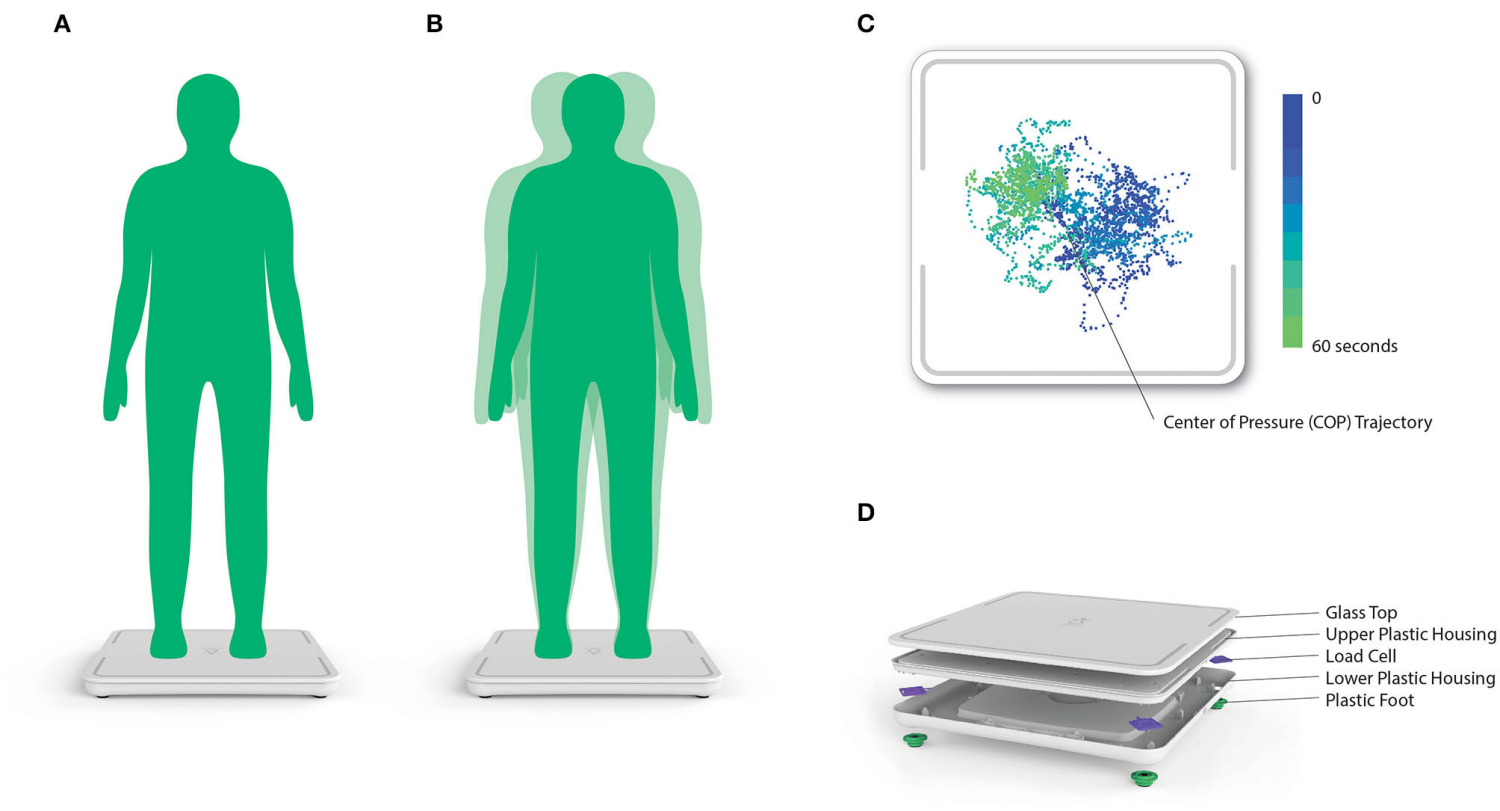
**(A,B)** Postural stability (PS) score testing setup, procedure and instructions. **(C)** Representative 2D center-of-pressure (COP) trace collected from the Zibrio SmartScale. The color gradient denotes the change in the COP trace over time throughout the 60 second standing balance test. **(D)** A 3D rendering of the components and assembly of the Zibrio SmartScale.

Upon enrollment, participants were instructed to stand still for 60 s, silently, with their arms to their sides, their feet comfortably shoulder-width apart and their eyes looking forward, on a commercially available SmartScale (Zibrio, Houston, TX, USA), see Figure 1. Participants were asked to wear their standard footwear during testing. This low-cost force plate was validated to accurately measure center-of-pressure (COP) over time with a frequency of 60 Hz (21). Using the collected COP data, linear quantifications of postural sway, including: path length, velocity, acceleration and jerk, in both anterior-posterior and medial-lateral directions, as well as non-linear measures of postural stability characterized using a Hidden Markov Model (19, 20) were utilized as factors (22) to calculate the PS score. The PS score is scored ranging from 1 to 10, where larger scores indicate higher stability. The parameters of the Hidden Markov Model were determined using COP data previously on a laboratory-grade force plate and no algorithmic refinement occurred during this study. No PS scores or balance feedback were provided to the participants. The above trial was immediately repeated. If technical and timing constraints did not interfere, a third test was also performed (in 322 cases). This allowed us to examine PS score reproducibility and participants were offered the opportunity to sit and rest between trials.

During an accompanying interview, participants reported age, medical condition status, and asked if they had experienced a fall in the past 6 months, a near fall in the past 6 months, a fall in the past 12 months, and a near fall in the past 12 months. These four ordered questions focused attention on a shorter time period first to optimize recall accuracy, while still extracting valuable longer period information (23). A fall event was counted if the participant confirmed that they had “unintentionally reached the ground or a lower level” (8), unless the event was due to self-reported orthostatic intolerance or syncope. Only one fall event from the initial interview was excluded, due to self-reported fainting.

For participants in the prospective study, monthly follow-up was performed via telephone, e-mail, or text-message for a year after initial testing, in order to document any changes in medical state and to collect reliable self-reports of fall or near-fall events (24). Participants were asked if they had experienced a fall in past month (or time since last communication), and then, if they had experienced a near fall in the past month (or time since last communication). Falls were classified as: (1) a slip or trip; (2) associated with a recent change in medical status (e.g., new medication, or a recent surgical procedure); (3) associated with a challenging movement or environment, i.e., “hiking while looking at a bird's nest”; or (4) a spontaneous fall, where no mitigating explanation could be provided. See Supplementary Table 1 for examples.

Follow-up efforts were uniform for all participants and continued throughout the 12-month period after enrollment. Occasionally an individual could not be reached, however, contact was attempted again the following month. Thirty individuals were considered lost-to-follow-up when contact could not be made after 3 months or the subject opted to withdraw from the study; in these censored cases the total enrolled duration will be <12 months (25). The PS score of participants lost-to-follow-up were distributed across PS scores 1–6, see Supplementary Table 2.

### Data Analyses

#### Postural Stability (PS) Score Test-Retest Reliability

We examined PS scores collected from all enrolled subjects (*n* = 412), including the 3-test series for 322 patients and the test pairs from 90 participants. Bivariate density plots were generated to highlight within-subject agreement across different trials and Pearson's correlations were calculated for these comparisons. We also calculated a single-measurement, absolute-agreement, 2-way random effects intra-class correlation [ICC(2,1)] (26) which included participants who completed at least two measurements (*n* = 380). Analyses of reliability were performed in R(v.3.6.1) (27).

#### Retrospective Fall Risk Analyses

We plotted cumulative PS score frequency distributions for subjects who reported falling in the 12 months prior to enrollment, as well as subjects who did not report falling in the 12 months prior to enrollment to explore the difference in PS score distributions between past-fallers and non-fallers. To further assess the relationship between PS score and fall history, we generated receiver-operating characteristic (ROC) curves and calculated the area under the curve (AUC). The slope of the retrospective ROC curve was observed to inform categorization of the PS scores into “high,” “moderate,” and “low” risk categories based on estimated likelihood ratios (LR) (28). Chi-squared analyses were performed to test for differences between “fallers” and “non-fallers” among each risk categorization.

#### Prospective Fall Risk Analyses

We plotted cumulative PS score frequency distributions for patients who fell during the 12-month follow-up period, as well as patients who did not fall during the 12-month follow-up period to explore the difference PS score distribution between prospective fallers and non-fallers. We also generated receiver-operating characteristic (ROC) curves and calculated the area under the curve (AUC). Chi-squared tests comparing PS scores for the three defined PS score risk categorizations were performed.

After subdividing the participants into three fall risk categories, fall-free survival analysis was performed using Log Rank and Cox proportional-hazard regression tests, including censored cases. Finally, the proportion of each fall cause, based on self-report during monthly follow-up, was calculated for each PS score risk category. Chi-squared analyses were performed to test for differences between risk categories.

## Results

### Participant Demographics

The community recruited participants enrolled in the study were typically younger, used fewer assistive devices and pharmaceuticals, and were less likely to have a positive fall history in the 12 months prior to enrollment relative to participants from independent senior living facilities, see Table 1. Participant demographics for the larger clinical trial cohort can be found in Supplementary Table 3.

**Table 1:** Participant demographics for community dwellers included in the retrospective and prospective fall risk study.

		**All community dwellers (% Total)**	**Community recruited (% CR)**	**Independent senior living residents (% iSLF)**
**Total participants**		**209**	**99**	**110**
Sex	Male	58 (27.8%)	37 (37.4%)	21 (19.1%)
	Female	151 (72.3%)	62 (62.6%)	89 (80.9%)
Age (years)		77.6 ± 0.8	67.8 ± 0.8	86.2 ± 0.6
BMI		25.49 ± 5.2	25.7 ± 5.1	25.3 ± 5.2
Assistive devices	None	169	96 (97.0%)	73 (66.4%)
	Walker	31 (14.8%)	1 (1%)	30 (27.3%)
	Cane	9 (4.3%)	2 (2%)	7 (6.4%)
4+ Medications	No	129 (61.7%)	80 (80.8%)	49 (44.5%)
	Yes	80 (38.3%)	19 (19.2%)	61 (55.5%)
Retrospective fallers	Non-fallers	136 (65.1%)	81 (81.8%)	55 (50%)
	Fallers	73 (34.9%)	18 (18.1%)	55 (50%)
Duration of follow-up		330 ± 5.2 days	303 ± 8.7 days	360 ± 3.4 days
Prospective fallers	Non-fallers	127 (61.2%)	65 (65.7%)	63 (57.3%)
	Fallers	81 (38.8%)	34 (34.3%)	47 (42.7%)
	New fallers	44 of 81	25 of 34	19 of 47
Postural Stability (PS) score		4.0 ± 0.14	5.1 ± 0.19	3.1 ± 0.17

### Reliability of the Postural Stability (PS) Score

**Figure 2:**
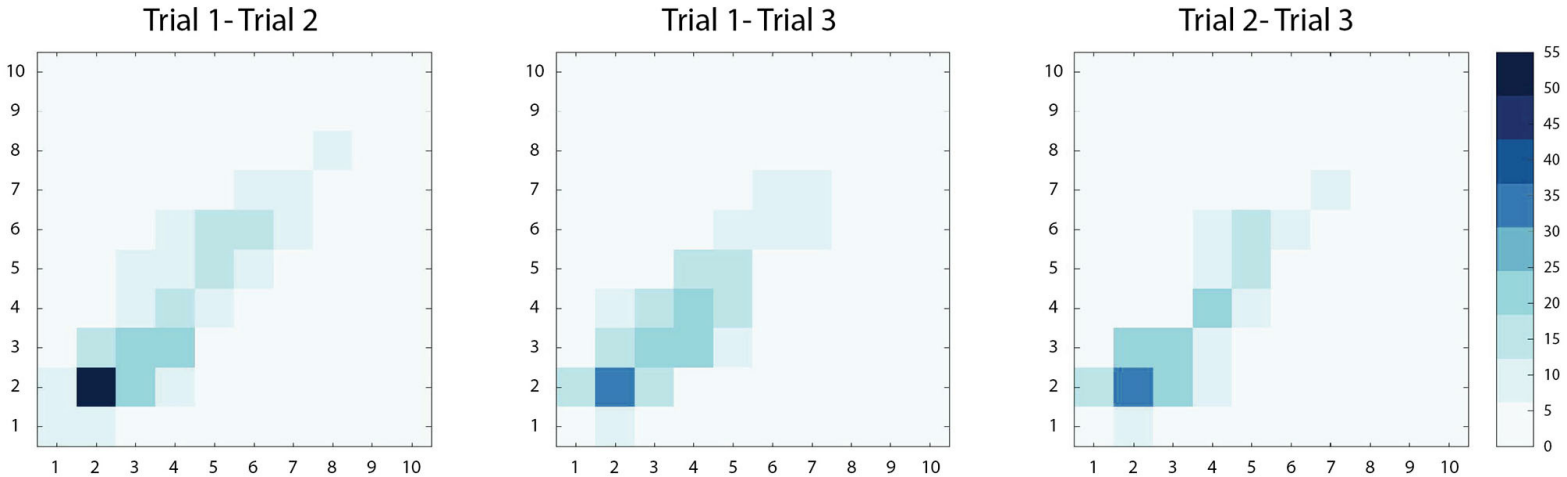
Bivariate density plots demonstrating within-subject agreement between two PS score measurements (scored 1–10). Increased agreement between two measures is demonstrated with increasing color intensity. Among the three comparisons, Trial 1-2, Trial 1-3, and Trial 2-3, the majority of paired observations lie along the diagonal, suggesting strong agreement. Across the diagonal, the greatest intensity is observed in the lower scores (PS score<5), where the majority of scores were observed in this study.

High correlations were observed among Trials 1-2 (*r*[95% CI] = 0.78 [0.74–0.82], *p* < 0.01), Trials 1-3 (*r*[95% CI] = 0.75 [0.70–0.79], *p* < 0.01) and Trials 2-3 (*r*[95% CI] = 0.82 [0.78–0.86], *p* < 0.01) implying good reliability among repeated measures. Bivariate density plots, highlighting the density and distribution of agreement between trials, are demonstrated in Figure 2. In general, the data exhibited a high level of test-retest reliability (ICC(2,1)[95% CI] = 0.78 [0.76–0.81]) (26) among the repeated measures taken at enrollment (*n* = 380).

### Retrospective Fall Risk Assessment

**Figure 3:**
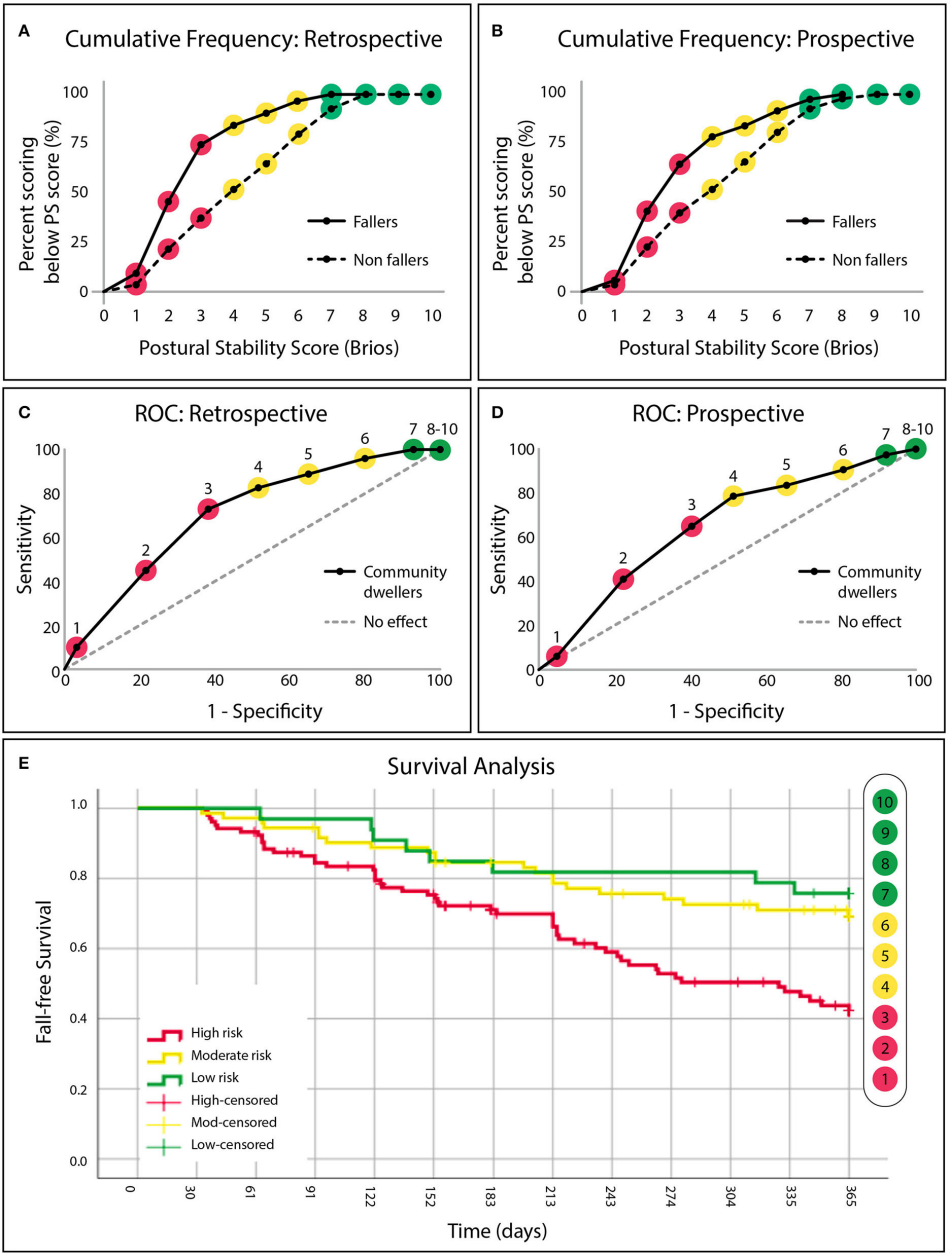
**(A)** The cumulative frequency distributions of the postural stability (PS) score for “fallers” and “non-fallers” identified from retrospective fall history and **(B)** prospective fall data. **(C)** Fall risk categories defined by receiver operating characteristic curves demonstrating the sensitivity and specificity of the PS score to identify community dwellers (independent senior-living residents or community recruited individuals) with a retrospective fall history. “High risk” (red, PS score: 1–3), “moderate risk” (yellow, PS score: 4–6), and “low risk” (green, PS score: 7–10) categories are defined by a change in slope. **(D)** Retrospectively defined risk categories applied to future falls observed in the same population after 12 months of longitudinal follow-up. **(E)** Cumulative survival curves denoting avoidance of a fall for all three risk categories (“‘high” in red, “moderate” in yellow and “low” in green), across 365 days (12 months).

Individuals with a history of falling exhibited systematically lower PS scores than those without a history of falling, see Figure 3A. PS scores of 1-3 had LRs (LR 3.3 to 1.7) to experience a past fall twice that of PS scores 4-6 (LR 0.7 to 0.5). LR halved again after PS scores of 7-10 (LR 0.3 to 0.0) which served to define the “high risk,” “medium risk,” and “low risk” categories, respectively. Individuals who were identified as “high risk” (PS score: 1-3) were significantly more likely to have experienced a fall in the past 12 months than those identified as either “low risk” (χ^2^ = 15.11, *p* < 0.01) or “moderate risk” (χ^2^ = 13.56, *p* < 0.01). Individuals identified as “moderate risk” were not found to be more likely to have experienced a fall in the past 12 months when compared with those identified as “low risk” (χ^2^=1.51). Classification as “high risk” by the PS score identified those with a positive fall history with 73.6% sensitivity, 62.8% specificity, see Figure 3C. The area under the ROC curve was 0.66.

### Prospective Fall Risk Assessment

Similar to the retrospective data, individuals who fell during the 12-month follow-up period exhibited systematically lower PS scores upon initial enrollment than those that did not fall, see Figure 3B. Individuals who were identified as “high risk” (PS score: 1-3) upon initial enrollment were 3.0 [1.4–6.3] (95% CI, *p* < 0.01) times more likely to fall during the 12-month follow-up period than those who identified as “low risk” (PS score: 7-10) (χ^2^ = 5.75, *p* < 0.01), and 2.2 [1.3–3.7] (*p* < 0.01) times more likely to fall than those identified as “moderate risk” (χ^2^ = 4.12, *p* < 0.01). Classification of “high risk” predicted that an individual would fall during the 12-month follow-up period with 64.2% sensitivity and 59.8% specificity. Area under the ROC curve was 0.64, see Figure 3D. Survival analysis revealed that the median time before an individual identified as “high risk” experienced a fall was 9.2 months, see Figure 3E. Individuals identified as “moderate risk” (PS score: 4–6) were not found to be more likely to fall during the follow-up period when compared with those identified as “low risk” (χ^2^ = 0.59).

**Figure 4:**
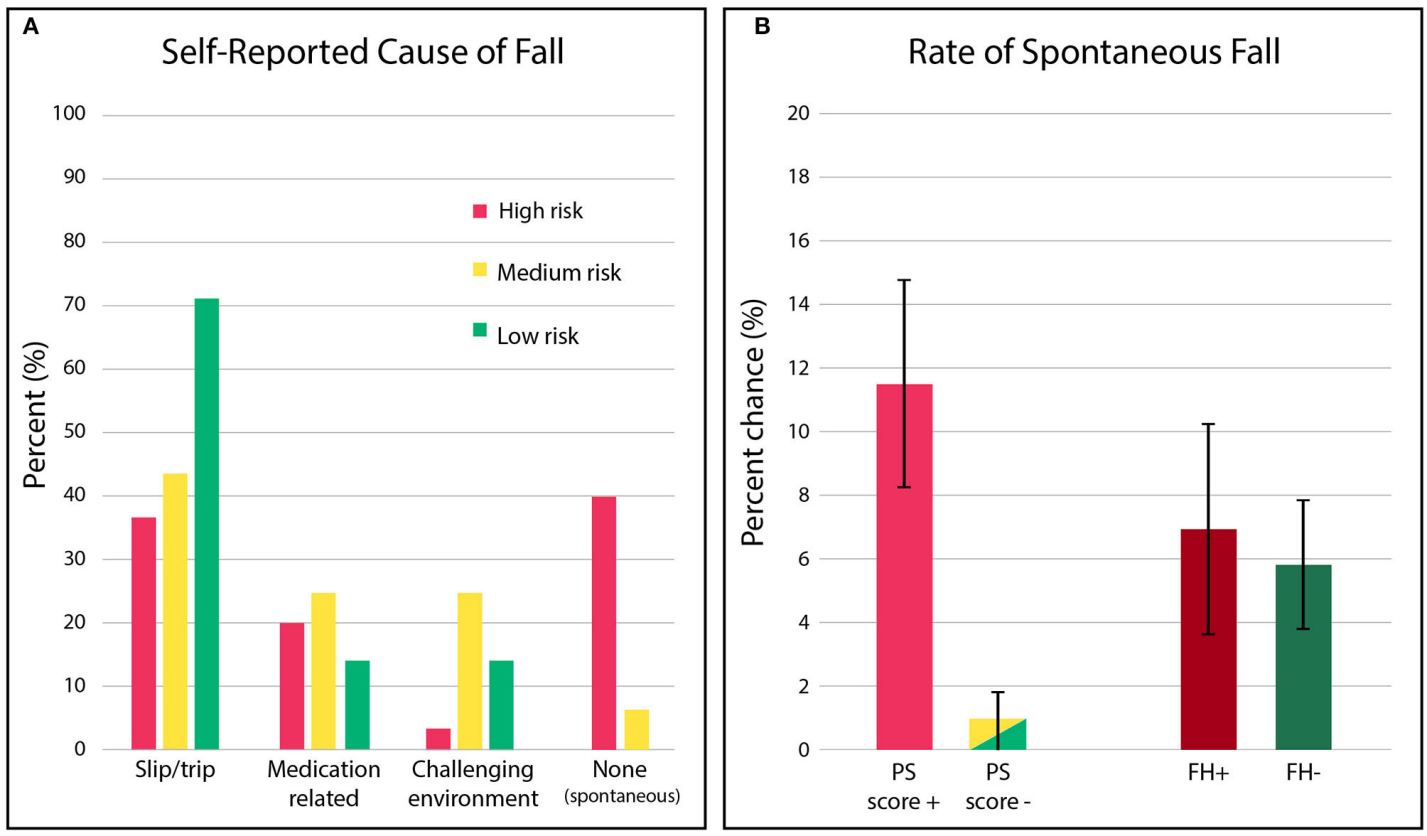
**(A)** Distribution of the fall causes observed in the prospective cohort, stratified by PS score risk categories. Red denotes “high risk,” yellow denotes “moderate risk,” and green denotes “low risk”. **(B)** The percent chance that individuals identified as “high risk,” based on postural stability (PS) score or fall risk (PS score+: 1-3 or FH+: history of falling), will experience a spontaneous fall (i.e., fall event where no mitigating cause was specifically reported) relative to individuals identified as “low” / “moderate risk” (PS score-: 4–10 or FH-: no history of a fall).

Strikingly, we observed a significant difference not only in the rate of falls among “high risk” individuals, but also in the kind of falls they suffered, see Figure 4A. Individuals classified as “high risk” were also an order-of-magnitude more likely (12 of 104 (11.5%) vs. 1 in 105 (1.0%), ~10× more likely) to suffer a spontaneous fall (i.e., one where no mitigating cause was identified, suggesting neither the environment nor changes in medical condition/medications were contributing factors).

By contrast, both individuals with (FH+) and without (FH-) a history of prior falls exhibited the same rate of spontaneous falling, see Figure 4B, suggesting fall history status provided no discrimination. Detailed results can be found in the Supplementary Table 4. In general, “low risk” fallers were most likely to fall due to trips/slips (72%), and “moderate risk” fallers were the most vulnerable to a fall while navigating a challenging environment (29%).

### Age Based Percentiles

**Figure 5:**
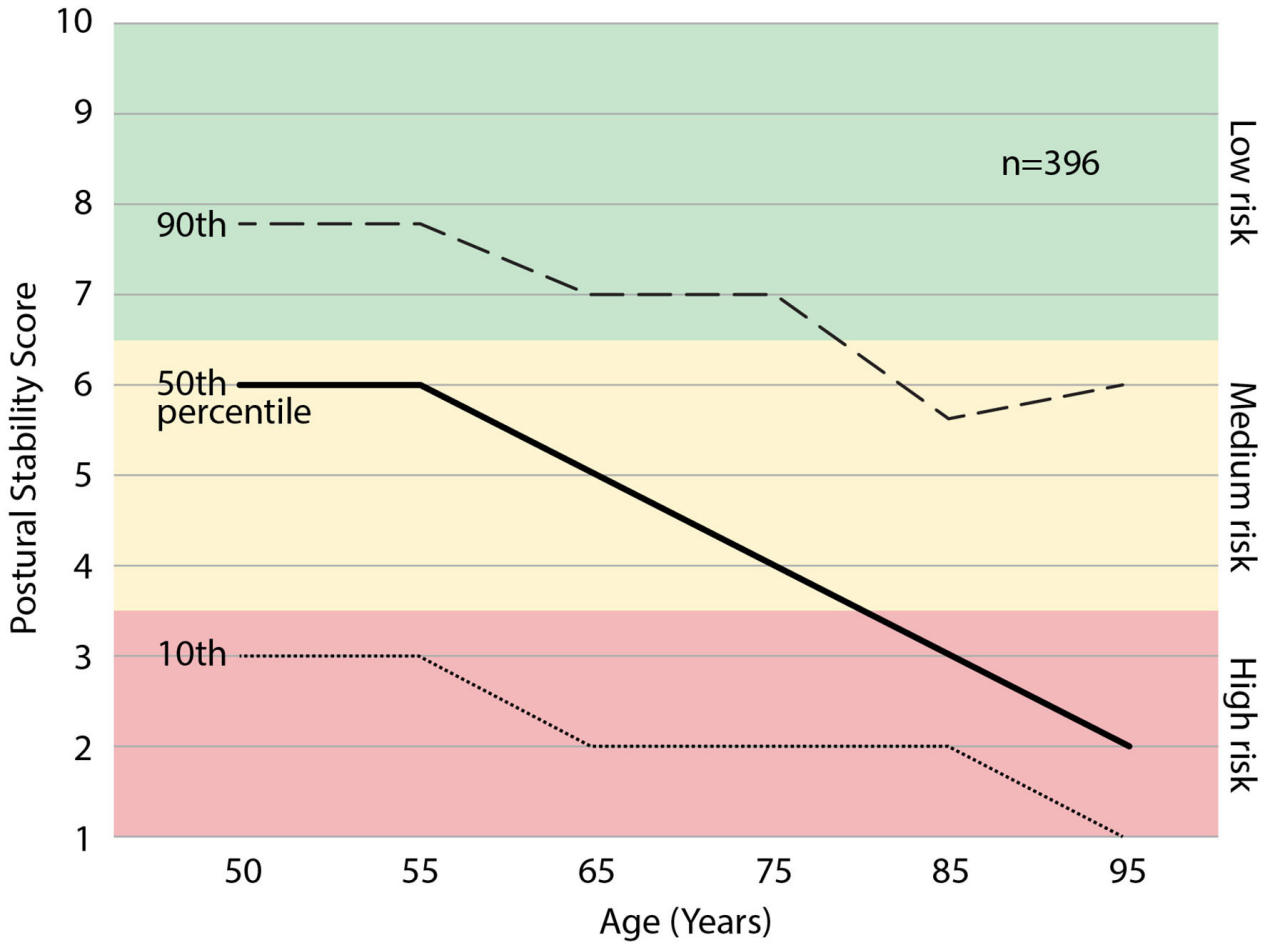
The percentiles of postural stability (PS) score with age. Those scoring in the 25th percentile may expect to enter the “high risk” category at ~65 yrs. and those 50th percentile may expect to enter the “high risk” category at ~80 yrs. of age.

We examined the relationship between age and PS scores within the sampled population, see Figure 5. Age bins were included if >10 participants were represented, resulting in a range of 50–95 yrs. Some individuals elected to not share age upon enrollment (*n* = 16), therefore the percentiles represent *n* = 396 of the enrolled individuals. Across all participants aged >50, the mean PS score declined with age across all percentiles, dropping ~1 point per decade. The 25th percentile crosses into the “high risk” category in the 60th decade, and the 50th percentile crosses into the “high risk” category in the 80th decade. The upper 25th percentile in the 90th decade (*n* = 36) exhibits a deviation from the deteriorating trend.

## Discussion

The study suggests that the postural stability (PS) score, generated with a machine learning method from a simple 60 sec., eyes-open, standing balance test is a reliable and valid method for identifying fall risk in aging adults, and predicts falls up to 12 months. Throughout the 12 months following enrollment, individuals categorized as “high risk” (PS score: 1–3) were 3.0 times more likely to fall than “low risk” individuals (PS score: 7–10) and 2.2 times more likely to fall than “moderate risk” individuals (PS score: 4–6). The predictive capability of the PS score is better than commonly used clinical tools as the prospective sensitivity for identifying a future “faller” from a “high risk” categorization is 64%, compared with 46% for fall history in this study, and 31% for TUG (29) and 15% for STEADI (30) from the literature. However, one third of fallers are not identified by PS score high risk categorization, and this suggests there is further opportunity for prediction improvement.

The PS score was especially strong at predicting spontaneous falls. Individuals identified as “high risk” (PS score: 1–3) had a 10× higher chance of experiencing a spontaneous fall. In this population, 92.3% of spontaneous fallers were identified as “high risk.” This dramatic effect indicates the use of the PS score could be an effective way to stratify individuals at risk of a fall for fall prevention counseling, insofar as the types of falls suffered by each group differ from one another. It is interesting, in this respect, to contrast PS scores with fall history, which is also known to be an indicator of future fall risk. Nevertheless, fall history did not predict whether an individual would have a spontaneous fall—there was no significant difference in the rate of spontaneous falling among those who did or did not have a history of falls. Taken together, our data is consistent with a model where spontaneous falls reflect intrinsic losses of stability, rather than falls caused by exogenous influences such as medication or the particular environment. If so, the advantage of a fall risk test that is sensitive to spontaneous falls is the capacity to identify patients with intrinsic balance instability issues that ought to be addressed clinically. Consequently, this test may facilitate precision fall prevention care for those whose underlying issues might otherwise go unnoticed.

We attribute the predictive advantages of the PS score to its ability to detect dynamic patterns of stability and instability (i.e., control failures) and reflect the capability of an individual's postural control system to minimize periods of instability. These measures are beyond the typical linear assessments of posturography and leverage new insights from machine learning and control systems theory (19, 20). As time-varying COP is readily available from any laboratory-grade force plate (21), this implementation of machine learning, which combines linear factors with the detection of primary control failures, can be applied to multiple populations at risk of postural control failure. This, in turn, enables risk to be determined before a fall history has been established and before deficits in functional performance are observed.

Although the classic Romberg standing balance test, utilizing both eyes-open and eyes-closed conditions is clinically used to seek out gross postural control deficits (31, 32), the sensitivity of the machine learning approach utilized in the present study makes the fall risk of eyes-closed standing balance unnecessary in at-risk populations. The same benefit vs. risk trade-off applies to other balance challenge tests such as the Clinical Test of Sensory Integration for Balance (CTSIB) which utilizes an unstable standing surface and requires an operator (33). By focusing on an innocuous condition of standing balance, this test is able to reduce operator burden and increase user safety, making it more accessible for fall risk management.

In general, the PS score was observed to decline with age, suggesting reduced postural stability control and increased fall risk with age. These data are in line with the U.S. national statistics that 1 in 4 over 65 years and 1 in 2 over 80 fall every year (2, 3). The corresponding 25th and 50th percentiles enter the PS score “high risk” categories at similar ages. In the ninetieth decade of age, there is a small upward trend in PS scores. This could be the result of those with poor balancing dying before reaching the ninetieth decade. Identifying a patient's PS score percentile in their age cohort can help patients to understand that a range of PS scores and fall risk exist at every age, and therefore, change is possible and improvements are attainable. The PS score percentile graph also illustrates the patient's fall risk trajectory. This means patient counseling can include future fall risk trajectories beyond the 12-month prediction window of their current fall risk categorization.

A common challenge for patient fall prevention counseling is patient denial or under-estimation of their own fall risk (34, 35), especially as a patient's own perception does not predict a fall (36). Thus, a simple, safe, objective, 60 sec. test with an easy to understand score, in the context of the patient's age cohort which identifies fall risk and future risk trajectory, may provide an easy way to remove barriers to enable effective patient counseling.

Falls have traditionally been considered unavoidable accidents. Yet our data suggests that, like routine patient discussions about hypertension, fall prevention counseling encompasses measurement, risk stratification, prevention by losing weight and exercise, and, when present, effective treatment. Different fall causes for each risk category indicate that the occurrence of a fall is the combination of a person's physical capability *and* his or her opportunity for falling. For example, a “high risk” person with poor stability control can sit in a chair all day and never fall, whereas a “low risk,” physically capable person may hike a treacherous trail while looking up at birds' nests and trip over a root (both of these scenarios occurred in the present study). Indeed, PS score “high risk” individuals fell spontaneously from being less physically capable (i.e., loss of stability), while PS score “moderate risk” individuals fell in more challenging environments.

As a result, some of the individuals identified as false positive may in fact be true positives with little opportunity to fall. Conversely, some false negatives may be higher performing individuals who engage in more risky behaviors. Both scenarios may explain why current clinical tools have such low predictive power for falls and why the PS score, despite having good predictive accuracy, also mis-categorized some individuals. To increase the PS score's predictive capability for all falls, future work can focus on enhancing prediction by accounting for individuals' opportunity to fall as a predictive factor. The independent senior living residents in the present study were, on average, older than the community recruited individuals and a higher percentage subsequently fell, 43 vs. 34%, respectively. These fall rates are in line with documented fall rates that increase with age (3, 8, 37). It is important, however, to acknowledge that different opportunities to fall may have existed for these two settings. A likely scenario is that fall rates were muted in the more protective environment of senior living as it is expected to have less opportunity to fall. Consequently, in this setting more false-positives would be anticipated, thus future work enhancing prediction by accounting for opportunity to fall should also consider age as a factor.

Given that opportunities to fall vary between individuals, the high PS score sensitivity to spontaneous falls provides confidence that a meaningful fall risk metric is being measured and can facilitate more personalized patient counseling. In practice, focus is typically placed upon “high risk” individuals, however risk identification can be just as important for “moderate” and “low risk” patients to get ahead of functional decline and achieve both fall prevention and a healthier population.

A limitation of this study is the lack of generalizability to people who were unable to stand unassisted for 60 s as well as individuals who self-reported a history of dementia, vestibular disorders (e.g., Meniere's disease), as they were excluded from the study. We suspect the risk for patients with Meniere's disease and vestibular disorders will be underestimated due to the protocol requirement of maintaining a still head. Future studies can look into the addition of controlled head movements to expose vestibular sensory weaknesses (38). People with dementia were excluded due to participant requirements of fall event recall in the study. If a dementia patient could adhere to the testing protocol, the results of this study may be applicable. Future work to confirm validity with dementia patients can include fall event confirmation from caregiver reporting. A further limitation to the generalizability of these results is the dependency of this assessment upon a commercial product, the Zibrio SmartScale, which may not be financially accessible to all clinical environments.

Self-reporting of falls has well-documented limitations and often results in underreported falls (24). While the present study aimed to reduce this limitation by optimizing event recall using short, monthly communications (23, 24), underreporting is still to be expected. Fortunately, injurious falls, the most relevant type of fall for public health, are most likely to be reported (24). Thus, despite reporting limitations, the findings from this study have significant relevance for public health and injury prevention. Emerging wearable technology that identifies fall events may be useful for addressing this limitation in future fall research.

The influence of participant demographics were not explored in detail in this study, therefore future work must strive to identify differences in PS scores among fall risk covariates such as sex (39, 40) and ethnicity (41). Given the insights from the present study, future work can also focus on different machine learning techniques to cluster force plate COP data for further resolution and prediction. A representative PS score is dependent on the user complying with the protocol of standing still without talking, moving their head, or fidgeting.

## Conclusions

The lack of a clear gold standard for clinical fall risk assessment (7), despite clinical guidelines (6), leaves aging and older patients underserved due to misleading and incomplete fall risk assessments. Misidentification of fall risk can lead to dramatic swings in clinical decisions, costs, and savings as the impact of falling is considerable. The present study demonstrates that a postural stability score, collected on a force plate and automatically generated from a 60 s, eyes open, standing balance test using machine learning techniques, can provide a reliable and valid method for identifying aging adults at-risk of falling.

## Data Availability Statement

The raw data supporting the conclusions of this article will be made available by the authors, without undue reservation.

## Ethics Statement

The studies involving human participants were reviewed and approved by Westerns IRB (#20171926 and #20172324) and University of Texas Health Science IRB (HSC-MS-16-0019). The patients/participants provided their written informed consent to participate in this study.

## Author Contributions

EL-A and SM were completely blinded to the data collection and only permitted to perform data analyses upon a deidentified, completed, dataset. KF aided by Kristin Bartlett, Andrea Case-Rogers, Jasmine Chigbu and Steven Wilberts collected all data in the assisted living, community dwelling, and physical therapy populations and was completely blinded to analyses until results were generated and compiled. A series of a priori analyses were performed to minimize investigator bias, as described in Supplementary Material 5. KW, SA, and NR from the University of Texas McGovern Medical School collected all data from hospital and clinical care facilities, and served as academic advisors in this study to ensure rigorous standards and manage conflicts of interest. All authors participated in the drafting and critical review of the manuscript prior to submission.

## Conflict of Interest

KF, EL-A, and SM are employed by Zibrio Inc. KW developed a conflict of interest ~12 months after completing testing due to her spouse becoming a private investor in Zibrio, Inc. The remaining authors declare that the research was conducted in the absence of any commercial or financial relationships that could be construed as a potential conflict of interest.
