# Supplementary material for: A Postural Assessment Utilizing Machine Learning Prospectively Identifies Older Adults at a High Risk of Falling
Source: Front Med (Lausanne). 2020 Dec 4;7:591517. doi: 10.3389/fmed.2020.591517 (PMC7772994; doi:10.3389/fmed.2020.591517)
Supplement: Supplementary file 1 [file Data_Sheet_1.docx]

Supplementary Material

Supplemental Table S1

Table S1: Examples of self-reported causes of fall events and their classifications

| **Fall Causes** | **Example from data records** |
| --- | --- |
| Spontaneous | “just fell”  “turned and fell”  “in garage, lost balance”  “lost balance in grocery store” |
| Slip or trip | “was doing yard-work, set down lawn edger, then tripped over the handle of it”  “fell while descending stairs, when reached bottom step, rushed and toe caught on carpet, causing subject to fall forward”  “lost balance in slippery wet driveway, was raining and possibly slick from oil”  “slipped on soapy water while mopping” |
| Change in medications or medical status | “recent knee replacement, learning to walk with walker”  “balance problem is caused by medications”  “couldn’t keep weight on leg any longer, had to go to hospital to get leg drained”  “started Prozac, but Prozac is making me feel dizzy”  “neural problems after stroke a month ago” |
| Risky behavior | “fell twice while hiking in the mountains”  “fell three times trekking chimps and gorillas in Uganda”  “fell on vacation in Germany, first night, did not turn on light, was jet-lagged and disoriented” |

Supplemental Table S2

Table S2: The distribution of postural stability (PS) scores for the 30 participants who were lost to follow-up. There was no potentially confounding bias observed across the range of PS scores representing this population.

| Postural Stability  (PS) Score | 1 | 2 | 3 | 4 | 5 | 6 | 7+ |
| --- | --- | --- | --- | --- | --- | --- | --- |
| # of participants  lost-to-follow-up | 5 | 2 | 6 | 5 | 5 | 5 | 0 |

Supplemental Table S3

Table S3: Detailed demographics of participants from the prospective cohorts presented in this manuscript.

|  |  | All (%) | Community recruited (% CR) | Independent senior living residents  (% iSLF) |
| --- | --- | --- | --- | --- |
| **Total Participants** |  | **209** | **99** | **110** |
| Sex | *Male* | 58 (27.8%) | 37 (37.4%) | 21 (19.1%) |
|  | *Female* | 151 (72.3%) | 62 (62.6%) | 89 (80.9%) |
| Age (years) |  | 82.6 ± 0.6 | 67.8 ± 0.8 | 86.2 ± 0.6 |
| BMI |  | 25.49 ± 5.2 | 25.7 ± 5.1 | 25.3 ± 5.2 |
| Assistive devices | *None* | 169 | 96 (97.0%) | 73 (66.4%) |
|  | *Walker* | 31 (14.8%) | 1 (1%) | 30 (27.3%) |
|  | *Cane* | 9 (4.3%) | 2 (2%) | 7 (6.4%) |
| 4+ Medications | *No* | 129 (61.7%) | 80 (80.8%) | 49 (44.5%) |
|  | *Yes* | 80 (38.3%) | 19 (19.2%) | 61 (55.5%) |
| Retrospective fallers | Non-Fallers | 136 (65.1%) | 81 (81.8%) | 55 (50%) |
|  | Fallers | 73 (34.9%) | 18 (18.1%) | 55 (50%) |
| Duration of follow-up |  | 330±5.2days | 303±8.7days | 360±3.4days |
| Prospective fallers | Non-Fallers | 127 (61.2%) | 65 (65.7%) | 63 (57.3%) |
| (*new fallers) | Fallers  *new fallers | 81 (38.8%)  *44 of 81 | 34 (34.3%)  *25 of 34 | 47/ (42.7%)  *19 of 47 |

Supplemental Table S4

Table S4: Distribution of spontaneous falls (falls where no cause was self-identified), stratified by postural stability (PS) score and fall history.

|  | Number of participants | # people who experienced a spontaneous fall | Percent chance of experiencing a spontaneous fall |
| --- | --- | --- | --- |
| PS score, low + moderate risk (PS score-) | 105 | 1 | 0.95 |
| PS score, high risk (PS score+) | 104 | 12 | 11.54 |
| Fall history, low risk (FH-) | 137 | 8 | 5.84 |
| Fall history, high risk (FH+) | 72 | 5 | 6.94 |

Supplemental Text S5

**Predefining Postural Stability (PS) score Prospective Analyses to Reduce Investigator Bias**

To minimize investigator bias, an investigator-blinded data collection and follow-up procedure was followed. Investigators who performed the balance testing were blinded to participant fall status during follow-up (i.e. reporting of fall, near-fall or no fall) and the investigators who performed participant follow-up were blinded to the PS score collected upon enrollment, therefore no individual on the team could know if reported fall events matched with a corresponding “high”, “moderate” or “low” risk PS score.

To reduce the risk of bias during data analysis an *a priori* set of analyses were performed to test the overall discriminating capability of the PS score to differentiate past “fallers” from “non-fallers” based on retrospective fall history. No reweighting or refinement of factors in the PS score calculation algorithm were performed at any time. A Chi-squared comparison of retrospective fall status relative to upper and lower PS score tertiles was selected. The goals of this procedure were: 1) to ensure an analysis strategy was defined by investigators blinded to the PS score testing, and 2) to ensure that a minimum effect size was detectable prior to proceeding with longitudinal follow-up.

We hypothesized that the upper and lower tertiles of the range of values observed in this population (top third of performers and lowest third of performers) would discriminate between the clinically meaningful classifications of past “faller” and “non-faller”. Based on the range of values observed, the upper tertile was represented by a PS score greater than 5 (PS score: 6-10, inclusive) and the lower tertile was represented by a PS score lower than 5 (PS score: 1-4, inclusive).

Overall, the PS score detected a clear, significant difference between past “fallers” and “non-fallers” (χ^2^=17.12 p<0.05). As a result, we determined the PS score to have potential discriminatory ability to identify future “fallers” and “non-fallers” and proceeded with longitudinal follow-up in the prospective cohort.

Upon completion of the longitudinal follow-up, the same, pre-defined analyses were performed comparing future “fallers” and “non-fallers” using the *retrospectively defined tertiles* providing the best opportunity to determine an unbiased assessment of future fall status discrimination using the PS score measurement. No reweighting or refinement of factors in the PS score calculation algorithm were performed at any time. The PS score detected a significant difference between future “fallers” and “non-fallers” (χ^2^=9.08 p<0.05). Only after confirming this discriminatory capability did we proceed with defining “high”, “medium”, and “low” risk categories based on the retrospective fall history and begin testing their discriminatory capability.
